# The nano- and meso-scale structure of amorphous calcium carbonate

**DOI:** 10.1038/s41598-022-10627-9

**Published:** 2022-04-27

**Authors:** Simon M. Clark, Bruno Colas, Dorrit E. Jacob, Joerg C. Neuefeind, Hsiu-Wen Wang, Katherine L. Page, Alan K. Soper, Philipp I. Schodder, Patrick Duchstein, Benjamin Apeleo Zubiri, Tadahiro Yokosawa, Vitaliy Pipich, Dirk Zahn, Erdmann Spiecker, Stephan E. Wolf

**Affiliations:** 1 School of Engineering, Macquarie University, Macquarie Park, NSW 2113 Australia; 2 Australian Centre for Neutron Scattering, Australian Nuclear Science and Technology Organisation, Locked Bag 2001, Kirrawee DC, NSW 2232 Australia; 3 Research School of Earth Sciences, The Australian National University, Canberra, ACT 2600 Australia; 4 Oak Ridge National Laboratory, Spallation Neutron Source, Pak Ridge, TN 37831 USA; 5 Rutherford Appleton Laboratory, Chilton, ISIS Facility, Didcot, Oxon OX11 0QX UK; 6 Department of Materials Science and Engineering (WW), Institute of Glass and Ceramics (WW3), Friedrich-Alexander-University Erlangen-Nuremberg (FAU), Martensstrasse 5, 91058 Erlangen, Germany; 7 Department of Chemistry and Pharmacy, Chair for Theoretical Chemistry / Computer Chemistry Centre (CCC), Friedrich-Alexander-University Erlangen-Nuremberg (FAU), Nägelsbachstrasse 25, 91058 Erlangen, Germany; 8 Institute of Micro- and Nanostructure Research (IMN) & Center for Nanoanalysis and Electron Microscopy (CENEM), Interdisciplinary Center for Nanostructured Films (IZNF), Friedrich-Alexander-University Erlangen-Nuremberg (FAU), Cauerstraße 3, 91058 Erlangen, Germany; 9 Jülich Centre for Neutron Science (JCNS), Forschungszentrum Jülich GmbH, Outstation at FRM II, Lichtenbergstrasse 1, 85747 Garching, Germany; 10 Interdisciplinary Center for Functional Particle Systems (FPS), Friedrich-Alexander University Erlangen-Nürnberg (FAU), Haberstrasse 9a, 91058 Erlangen, Germany

**Keywords:** Nanoscale biophysics, Biomineralization, Glasses

## Abstract

Understanding the underlying processes of biomineralization is crucial to a range of disciplines allowing us to quantify the effects of climate change on marine organisms, decipher the details of paleoclimate records and advance the development of biomimetic materials. Many biological minerals form via intermediate amorphous phases, which are hard to characterize due to their transient nature and a lack of long-range order. Here, using Monte Carlo simulations constrained by X-ray and neutron scattering data together with model building, we demonstrate a method for determining the structure of these intermediates with a study of amorphous calcium carbonate (ACC) which is a precursor in the bio-formation of crystalline calcium carbonates. We find that ACC consists of highly ordered anhydrous nano-domains of approx. 2 nm that can be described as nanocrystalline. These nano-domains are held together by an interstitial net-like matrix of water molecules which generate, on the mesoscale, a heterogeneous and gel-like structure of ACC. We probed the structural stability and dynamics of our model on the nanosecond timescale by molecular dynamics simulations. These simulations revealed a gel-like and glassy nature of ACC due to the water molecules and carbonate ions in the interstitial matrix featuring pronounced orientational and translational flexibility. This allows for viscous mobility with diffusion constants four to five orders of magnitude lower than those observed in solutions. Small and ultra-small angle neutron scattering indicates a hierarchically-ordered organization of ACC across length scales that allow us, based on our nano-domain model, to build a comprehensive picture of ACC formation by cluster assembly from solution. This contribution provides a new atomic-scale understanding of ACC and provides a framework for the general exploration of biomineralization and biomimetic processes.

## Introduction

A molecular understanding of the processes that drive mineral formation is essential for deciphering biomineralization processes^1–6^, accurate assessment of paleoclimate records^7–9^, control of mineral formation^10,11^ and to accelerate the development of biomimetic materials^12,13^. Transient amorphous precursors such as amorphous calcium carbonate (ACC) play a crucial role in both biological and synthetic mineralisation systems. A knowledge of the atomic structures of these intermediates is a prerequisite for fully elucidating formation pathways, identifying biological control mechanisms and for understanding the role of polyamorphic^14^ phase transformations in determining final crystalline products. These are, however, difficult to determine unambiguously due to the transient nature of these materials and a lack of long-range order. Three recent studies of synthetic and non-deuterated ACC used Monte Carlo simulations^15^ constrained by scattering data to address this issue but produced disparate pictures. The first study by Goodwin et al.^16^, using ACC generated without stabilizing additives, yielded a structural model of a nanoporous charge-separated calcium-rich framework pervaded by interconnected channels rich in water and carbonate. In contrast, two other studies using Mg-doped^17^ and non-doped ACC^18^ proposed a homogenous structure with no evidence for long-range hydrogen-bonded networks. In order to resolve these conflicting models we used a recently developed method to synthesise a deuterated, non-doped ACC sample which is stable for many days^19^ and which allowed us to collect neutron and X-ray scattering data of unprecedented quality (Supplementary Fig. S1). These data, combined with Monte Carlo simulations^15^ and model building produced, for the first time, a detailed atomic structure for ACC. Transmission electron microscopy (TEM) and selected area electron diffraction (SAED) further supported our conclusions while small angle neutron scattering (SANS) extended our study up to the micron lengthscale. Molecular dynamics simulations allowed us to asses the physical properties and to substantiate the chemical (meta)stability of our novel ACC model.

## Results

**Figure 1:**
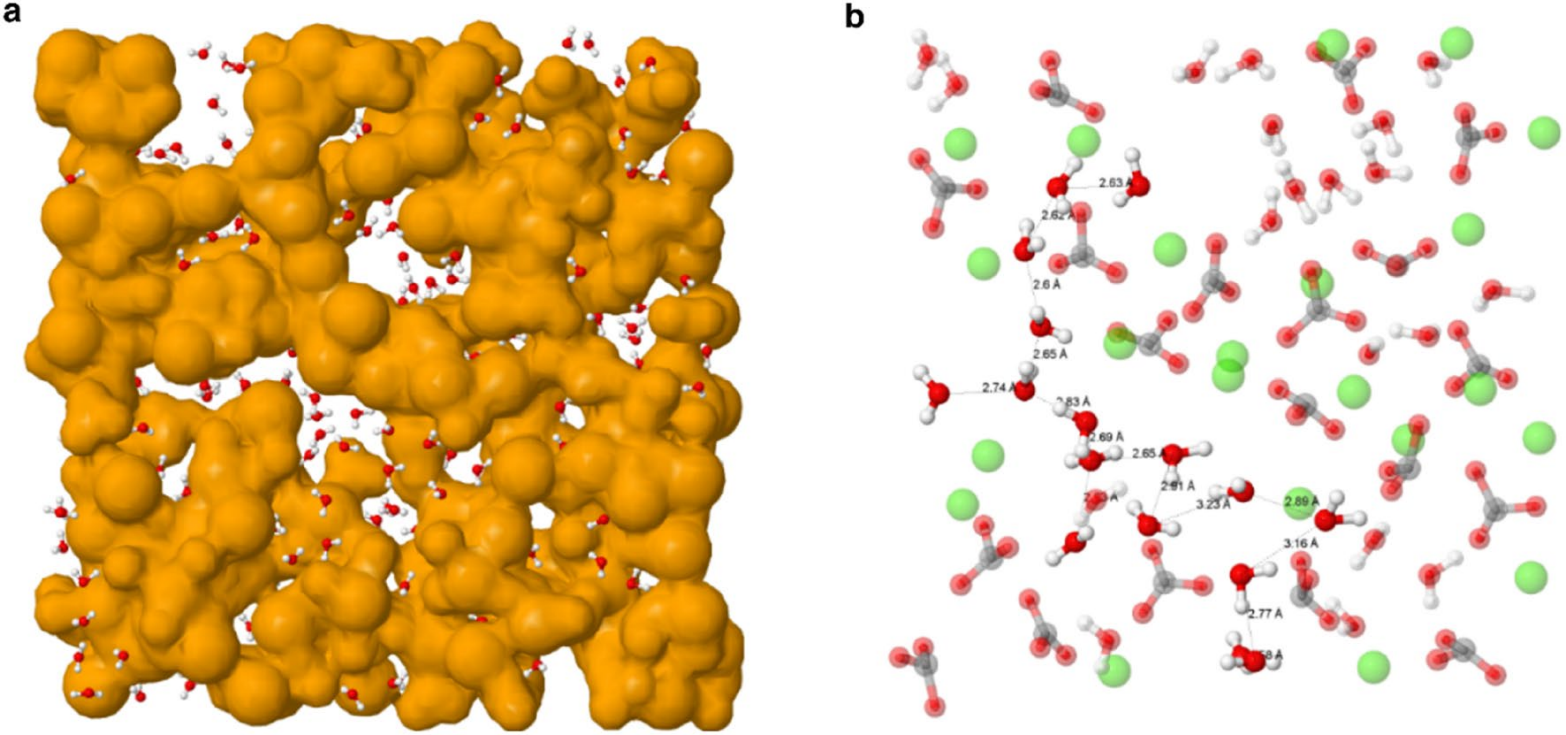
Projections of the ACC structure model produced by Monte Carlo simulations constrained by X-ray and neutron scattering data. (**a**) Calcium atoms are represented by oversized mustard-coloured spheres and water molecules by a ball and stick model with red and white spheres representing the oxygen and hydrogen atoms, respectively. The carbonate groups are contained within the spheres centred on the calcium atoms. Calcium carbonate units form anhydrous clusters; water is distributed around the surface of these clusters with no substantive regions of bulk water. (**b**) Expanded view of subfigure a with calcium atoms shown as green spheres, carbon as grey spheres and oxygen as a red sphere and water molecules as a ball and stick model with oxygen as a red sphere and hydrogen as a white sphere. Water molecules form chains around anhydrous calcium carbonate clusters. One chain has been highlighted to demonstrate a typical water molecule arrangement with a principal chain and periodic branching. The oxygen–oxygen intermolecular distances are shown along the chain.

The neutron and X-ray scattering data used in this work are shown in Supplementary Fig. S1 together with the model fit to these data. The model was produced by first carrying out Monte Carlo simulations^15^ constrained by the data which produced sets of atomic coordinates from which average pair-distribution functions and coordination numbers as well as bond and torsion angle probability distributions were determined (Supplementary Figs. S2, S3). These probability distributions feature sharp peaks characteristic of a high degree of short-range order within the nano-domains indicative of crystalline structures. Analysis of these probability distributions using a model building approach revealed a structure composed of highly ordered anhydrous nano-domains of around 2 nm (Fig. 1a) with an interstitial net-like matrix of water molecules (Fig. 1b) arranged to give a heterogeneous structure on the mesoscale (Supplementary Fig. S4, Supplementary Table S1).

The X-ray scattering data presented here (Supplementary Fig. S1) and in the previous structural studies of ACC are very similar with peak intensities at about 2.22, 3.14, 5.36, 6.23 Å^−1^ (Supplementary Table S2). This might be expected since the differences between the samples (substitution of 5% of the Ca atoms by Mg in the study of Cobourne et al.^17^ and varying amounts of water) would only cause very slight changes to the X-ray scattering intensity. The neutron scattering patterns presented by us, by Cobourne et al.^17^, and by Jensen et al.^18^ are also similar. All peaks in these scattering patterns above and including the 3.2 Å^−1^ peak in our data, and the equivalent 2.98 Å^−1^ and 3.15 Å^−1^ peaks in the Cobourne et al.^17^ and Jensen et al.^18^ data, have similar relative intensities. The major difference between the data presented in these three studies is found in the low Q region. The 2.22 Å^−1^ peak in our data is much more intense than all the other peaks in our scattering pattern while the equivalent peaks at 1.84 Å^−1^ in the Cobourne et al.^17^ data and at 1.99 Å^−1^ in the Jensen et al.^18^ data have similar intensities to all other peaks in those patterns. This difference is probably due to the incoherent scattering from the hydrogen atoms in the samples used by Cobourne et al.^17^ and Jensen et al.^18^ reducing the peak intensity compared to the deuterated sample used in our study which scatters coherently. The differences in the peak positions are probably due to the differences in scattering between H_2_O and D_2_O and Mg–O and Ca–O. The only obvious difference between the neutron scattering data presented by Jensen et al.^18^ for the samples containing 0.5 and 1.1 molecules of water per carbonate molecule is a small increase in the intensity of the peak at 2.98 Å^−1^.

**Figure 2:**
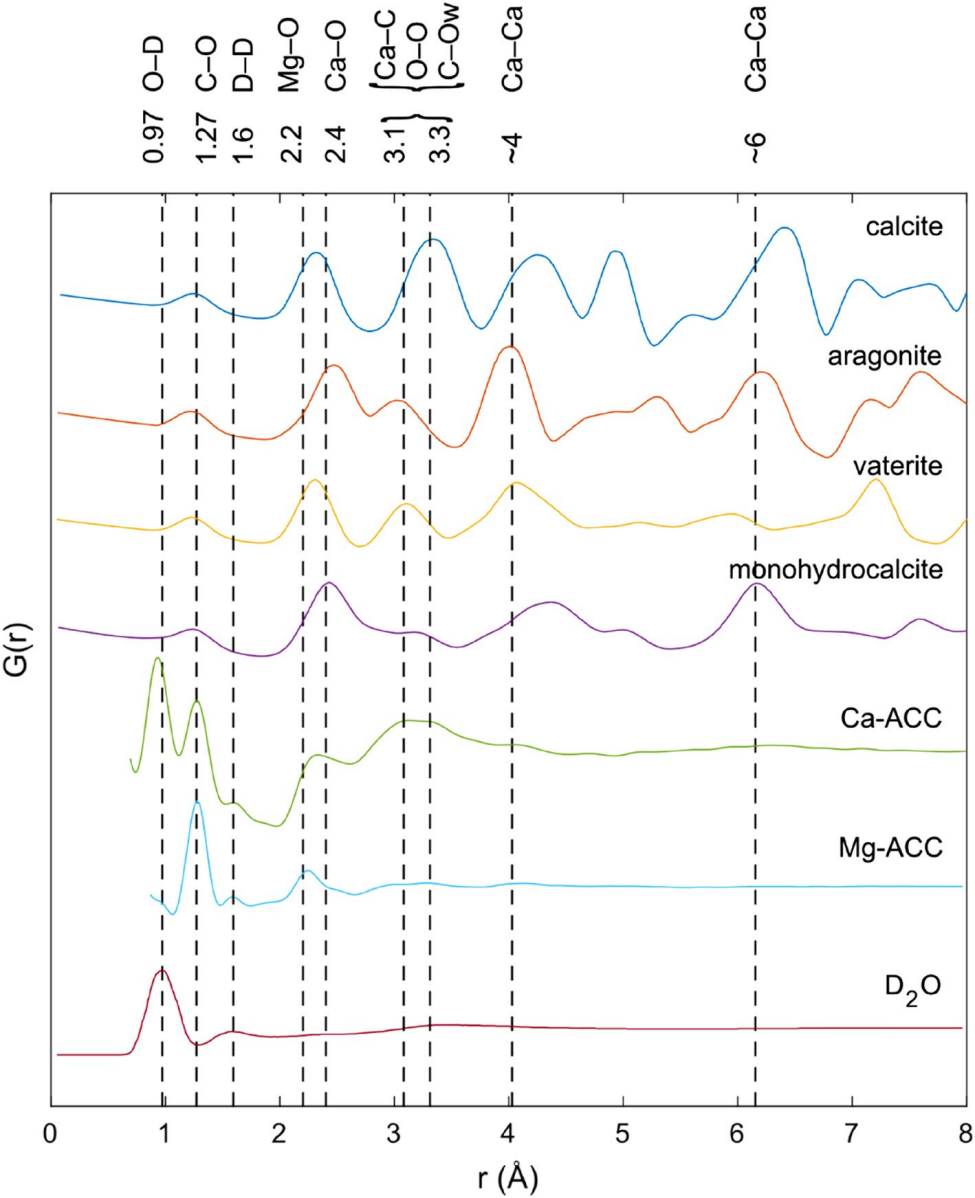
Comparison of the experimental total pair distribution functions for all known hydrous and anhydrous phases of calcium carbonate, i.e., calcite, aragonite, vaterite and monohydrocalcite^14^, pure ACC studied in this work, Mg-stabilised ACC (from Cobourne et al.^17^) and pure heavy water (Soper et al.^49^). The dashed lines show the positions of significant pair correlations. The total pair distribution function of monohydrocalcite contains no intramolecular H2O correlations due to it being determined from X-ray scattering data which is unable to detect these correlations.

The total pair distribution functions (PDFs, see Fig. 2) reported in these studies are also similar with maxima at about 1.27 Å, primarily due to intramolecular C–O correlations, 2.4 Å, primarily due to Ca-O correlations, 3.1 Å and 3.3 Å, primarily due to a combination of Ca–C, O–O and C–Ow correlations, where Ow refers to an oxygen that is part of a water molecule, and 4 Å and 6 Å primarily due to Ca–Ca correlations (Fig. 2, Supplementary Table S3). The partial pair distribution functions (pPDFs) for the Ca-ACC models derived from the Monte–Carlo simulations presented in these studies display quite consistent heavy atom correlations (Supplementary Table S4). For example: Ca–Ca maxima occur at about 4 Å, 6 Å and 8 Å, Ca–O maxima occur at about 2.4 Å, 4.1 Å, 6.2 Å and Ca-C maxima occur at 2.8 Å, 3.3 Å and 6.5 Å. The two studies that used a Mg-free sample and combining neutron and X-ray data (i.e., this work and Jensen et al.^18^) both report correlations between various atoms and hydrogen. These correlations again are similar with, O–H maxima at about 3.2 Å and 5.1 Å and Ow–H maxima at about 2 Å, 2.9 Å, 3.2 Å and 5.6 Å. The main difference between the two sets of results is that the first O–H correlation occurs at 1.8 Å in Jensen et al.^18^ and at 2 Å in this work. The distribution of coordination numbers for Oc (an oxygen that is part of a carbonate group) about Ca and Ow about calcium were found to be the same in both studies. The Mg-stabilised sample studied by Cobourne et al.^17^ shows some similarities to the ACC samples with, for example, maxima in the Ca–O correlation at 2.4 Å and in the Ca–C correlation at 3.29 Å and 6.1 Å (with just the 2.8 Å peak missing) but poor agreement for Ca–Ca correlation with maxima at about 3.44 Å and 6.141 Å as opposed to 4 Å, 6 Å and 8 Å in the Mg free samples and maxima in the C–O pPDF with a maximum at 2.6 Å as opposed to maxima at 3.8 Å and 5.1 Å in the Mg free samples. The full width at half maximum (FWHM) for these peaks are of the same order as that found for the Mg-free samples suggesting that the Mg doped samples are also made up of crystalline nanoparticles although the smaller values for the maxima in the Ca–Ca and C–O would suggest the that Mg doped sample has a different atomic structure and is denser. Thus, it is significant that the model that we present here is consistent with all the scattering data and simulation results considered above despite the varying compositions and synthesis paths of the samples used in the various studies suggesting the existence of a single, unique ACC atomic structure.

**Figure 3:**
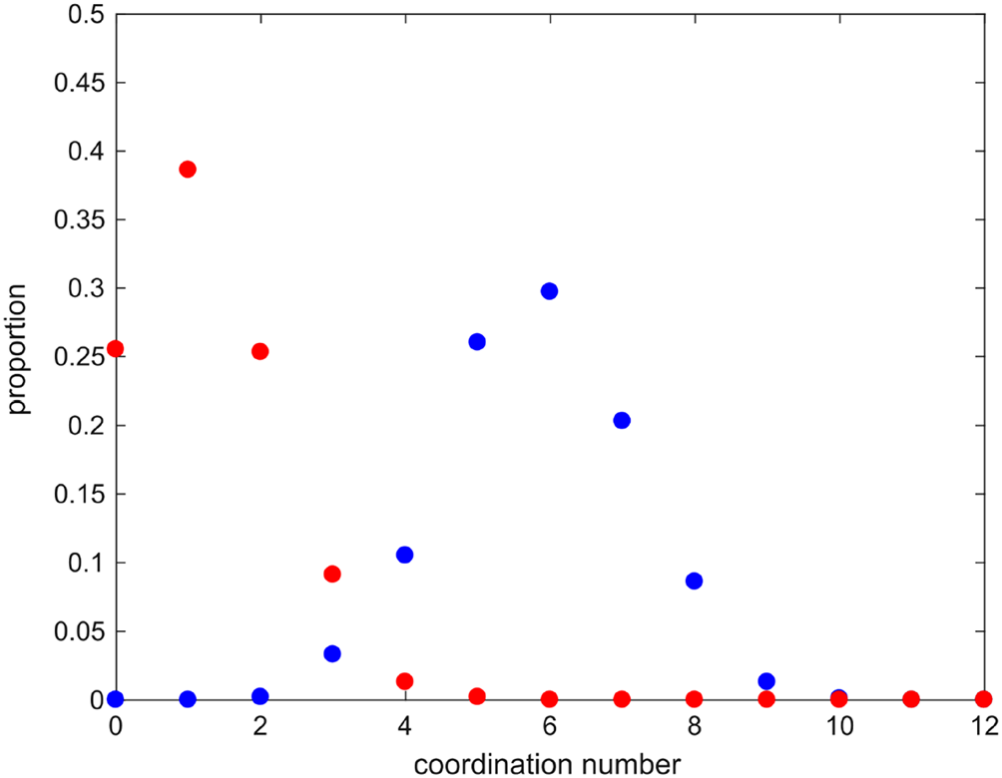
Proportion of oxygen atoms found within 3 Å of a calcium atom for: (blue filled circle) oxygen atoms in carbonate and (red filled circle) oxygen atoms in water molecules.

The proportion of oxygen atoms found within 3 Å of a calcium atom are given in Fig. 3, discriminating oxygen atoms in carbonate and oxygen atoms in water molecules. On average, calcium atoms are surrounded by six oxygens from carbonate groups and one oxygen from a water molecule with about 26% of calcium atoms not associated with a water molecule. A spherical ACC nanoparticle of 2 nm size has a volume of 2.3 nm^3^. This is equivalent to 36 unit cells of our ACC model. If we arranged these in a 4 × 3 × 3 array, then there would be 68 calcium atoms on the surface and 12 in the interior. Only the (110) and (011) faces have water associated with them (see Supplementary Fig. S5). Not all calcium atoms on a specific plane can have water molecules associated with them since there are no 4, 6 or 8 Å correlations in the Ow–Ow partial pair distribution function. Assuming one water molecule associated with every other unit cell results in a total of 20 unit cells with a water molecule associated. On average 20 out of the 80 calcium atoms in a given nano-cluster (or 25%) are associated with oxygens in water molecules.

X-ray and neutron scattering allow to determine whether a material is amorphous or consists of a collection of nanoparticles since the later will have a large rise in scattering at low Q due to heterogeneities which are absent for the former. Distinguishing between amorphous or crystalline nanoparticles is not as straight forward since there are no long-range correlations, due to the small particle size. In addition, any surface distortions to the atomic positions will have a relatively large impact on the overall average structure, since there are many more atoms on the surface than in the bulk, thus blurring the distinction between crystalline and amorphous. Comparison with other amorphous systems might help us decide how crystalline or amorphous ACC really is. Ca_0.4_K_0.6_(NO_3_)_1.4_ (CKN) is a simple ionic glass^20^ that contains NO_3_^−^ molecules and is governed by Columbic interactions and is therefore similar to ACC. The sharp peaks in the partial pair distribution functions of CKN (see Fig. 7 in Tengroth et al.^20^) for N–O and O–O are due to intramolecular correlations in the NO_3_^−^ group. These have FWHM of about 0.1 Å and 0.2 Å respectively which are comparable with the C–O correlation FWHM of 0.1 Å found in our ACC structural model (Supplementary Fig. S2). The Ca–Ca, and the related K–Ca and K–K correlation FWHM in CKN are about 2 Å and all other correlation FWHM, including O–Ca, are about 1 Å (see Fig. 7 in Tengroth et al.^20^). This has to be compared with a Ca–O correlation FWHM of about 0.23 Å in our ACC structure (Supplementary Fig. S2). This demonstrates that the molecular component of our ACC structure has a similar degree of ordering to a molecule in a typical ionic glass while the Columbic part of our ACC structure is significantly more ordered than that of a typical ionic glass. Another appropriate comparison is with amorphous silica (aSiO_2_) which exists as a polymeric network of corner shared SiO_4_ tetrahedra (or molecules for the sake of this discussion) with a range of Si–O–Si intermolecular angles and Si–O–Si–O intermolecular torsion angles. This results in an overall structure having no long-range order but nevertheless is more ordered than a typical Columbic glass such as CKN. The aSiO_2_ intramolecular distances and angles are reasonably well constrained about normal tetrahedral values^21^ (for example: O–Si–O has a FWHM of 10°) while the aSiO_2_ intermolecular angles are more dispersed (for example: Si–O–Si has a FWHM of 25°). In our ACC model (Supplementary Fig. S3) all of the bond angle distributions are nearer to the aSiO_2_ intramolecular FWHM than the intermolecular FWHM. For example: O–Ca–O has a FWHM of 10°, Ca–O–Ca has a FWHM of 10°, Ca–O–C has a FWHM of 12°, Ca–Ca–Ca has a FWHM of 15° and even Ow–Ca–Ow has a FWHM of only 12°. So if the intramolecular aSiO_2_ angles are relatively ordered and the intermolecular aSiO_2_ angles are relatively disordered then our ACC model is more ordered than aSiO_2_ with regard to angular distributions. The bond length FWHM in our ACC model do at first sight appear to be larger than those in aSiO_2_ (aSiO_2_ intramolecular Si–O FWHM 0.05 Å and intermolecular Si–Si FWHM 0.2 Å compared to ACC intramolecular C–O FWHM 0.1 Å and Columbic Ca–O FWHM 0.23 Å), but calcium in ACC exhibits a range of coordination numbers (with different bond lengths) and the carbonate molecule in ACC is known to exist with various degrees of non-planarity and other distortions^22^ while the SiO_4_ tetrahedron in aSiO_2_ is highly regular. These considerations lead us to conclude that ACC is probably closer to a disordered nano-crystalline material than to a fully amorphous nano-particular material. That this is the actual structure of ACC and not an artifact induced by our laboratory preparation is already attested by TEM observations of lattice fringes in biogenic ACC^23^ that match the spacings between high electron density planes in our ACC structure. Our findings confirm an earlier suggestion by Rez et al.^24^ who proposed that the coherent diffraction profile of ACC was due to randomly oriented crystallites of about one nanometre in diameter. Our data confirm that ACC indeed can be described as a cryptocrystalline modification of calcium carbonate hydrate. Although ACC with this ultrastructure is nano-crystalline on an atomic length scale it nevertheless still complies with the general definition of an amorphous material which requires either long-range order or sufficiently sharp diffraction peaks, both of which are absent for the case of ACC^25^.

**Figure 4:**
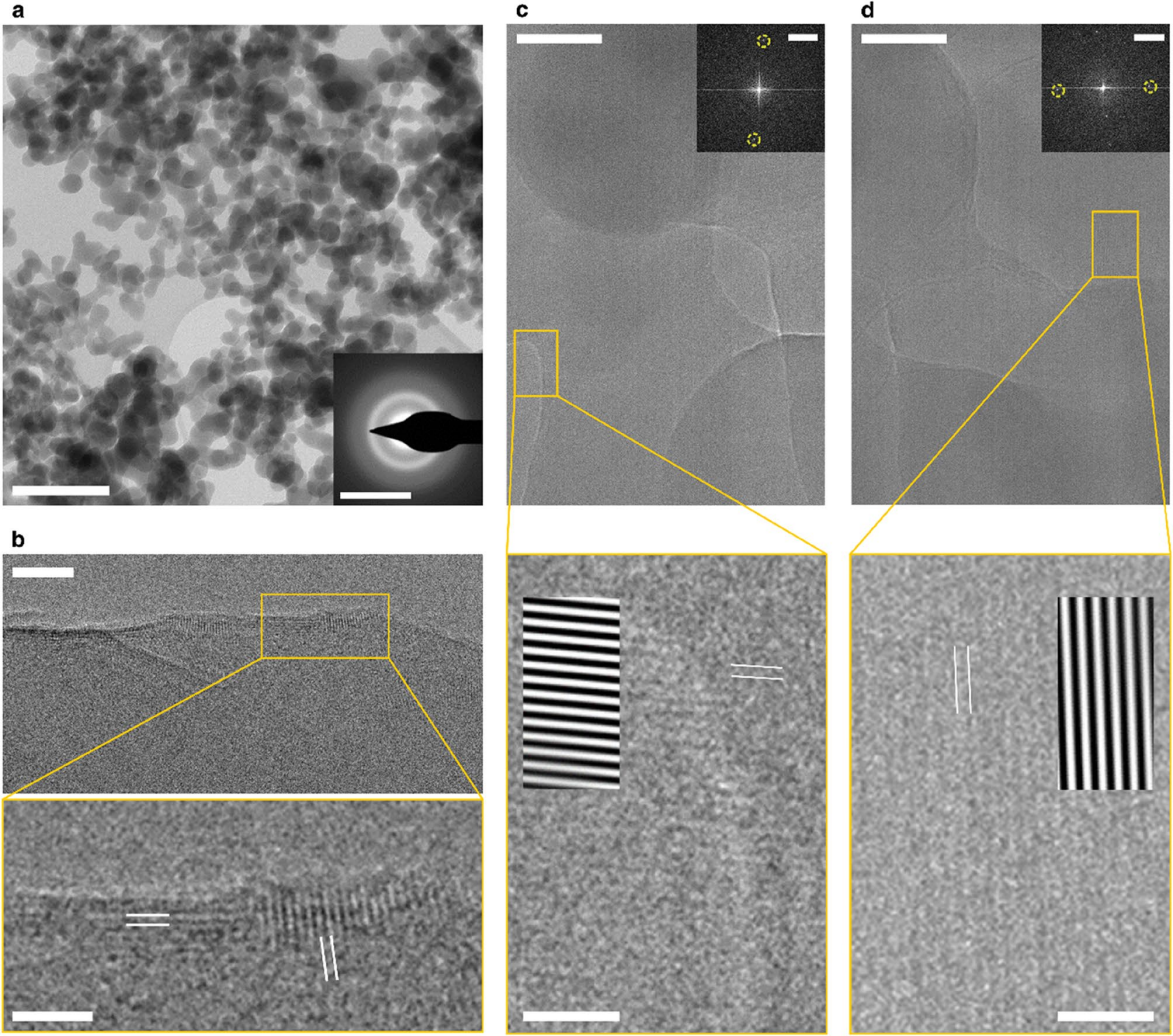
Transmission electron microscopy (TEM), selected area electron diffraction (SAED), and fast Fourier transform (FFT) analyses of amorphous calcium carbonate precipitated at high pH. (**a**) Bright-field TEM overview image of the ACC structure dispersed on a Lacey carbon support grid (scale bar: 200 nm). The inset shows a SAED ring pattern indicating arbitrarily oriented crystallites (scale bar: 5 nm^−1^), with diffuse peaks at 3.549 nm^−1^ and 5.315 nm^−1^ corresponding to real space distances of about 0.28 nm and 0.18 nm respectively. (**b**) High-resolution (HR) TEM micrographs of an edge region of the ACC nanoparticles (top scale bar: 5 nm, bottom scale bar: 2 nm), which had been irradiated with as little electron dose as possible, showing crystalline regions with a few nm in size with a lattice spacing of 0.24 nm, as indicated with white lines. (**c,d**) HR-TEM images of ACC after further electron beam irradiation; larger crystalline regions of 10–30 nm in size form (top scale bar: 20 nm); the bottom micrographs are enlarged views of the marked regions (bottom scale bar: 2 nm). The upper insets represent FFTs of the upper micrographs (scale bar: 2 nm^−1^). In the lower and enlarged micrographs, the banded areas are FFT-filtered areas using the FFT signals accentuated by yellow rings. The observed lattice spacings in subfigures (**c,d**) can be assigned to calcite (d_104_ = 0.30235 nm) and vaterite (d_−312_ = 0.32983 nm), respectively.

Bright-field TEM data and electron diffraction analyses provide additional experimental corroboration of our nano-structural model (Fig. 4). The selected area electron diffraction (SAED) pattern of pristine ACC consists of diffuse rings at 3.549 nm^−1^ and 5.315 nm^−1^. These correspond to the first two peaks in our X-ray diffraction data (Supplementary Fig. S1)^4,24,26^. A Scherrer analysis of the SAED patterns gave a mean particle size of about 1.8 nm which agrees well with the two-nanometre sized nano-domains determined from our Monte–Carlo-simulations. High-resolution TEM analyses of an edge area detected crystalline regions with a lateral diameter of about one to two nanometres which appeared during irradiation (Fig. 4b). Further irradiation led to the formation of more extended crystalline regions of up to 30 nm with mixed lattice spacings, suggesting that they contain both calcite and vaterite (Fig. 4c,d) demonstrating a direct connection between our cryptocrystalline ACC and the crystalline products observed in nature^14,26–29^.

**Figure 5:**
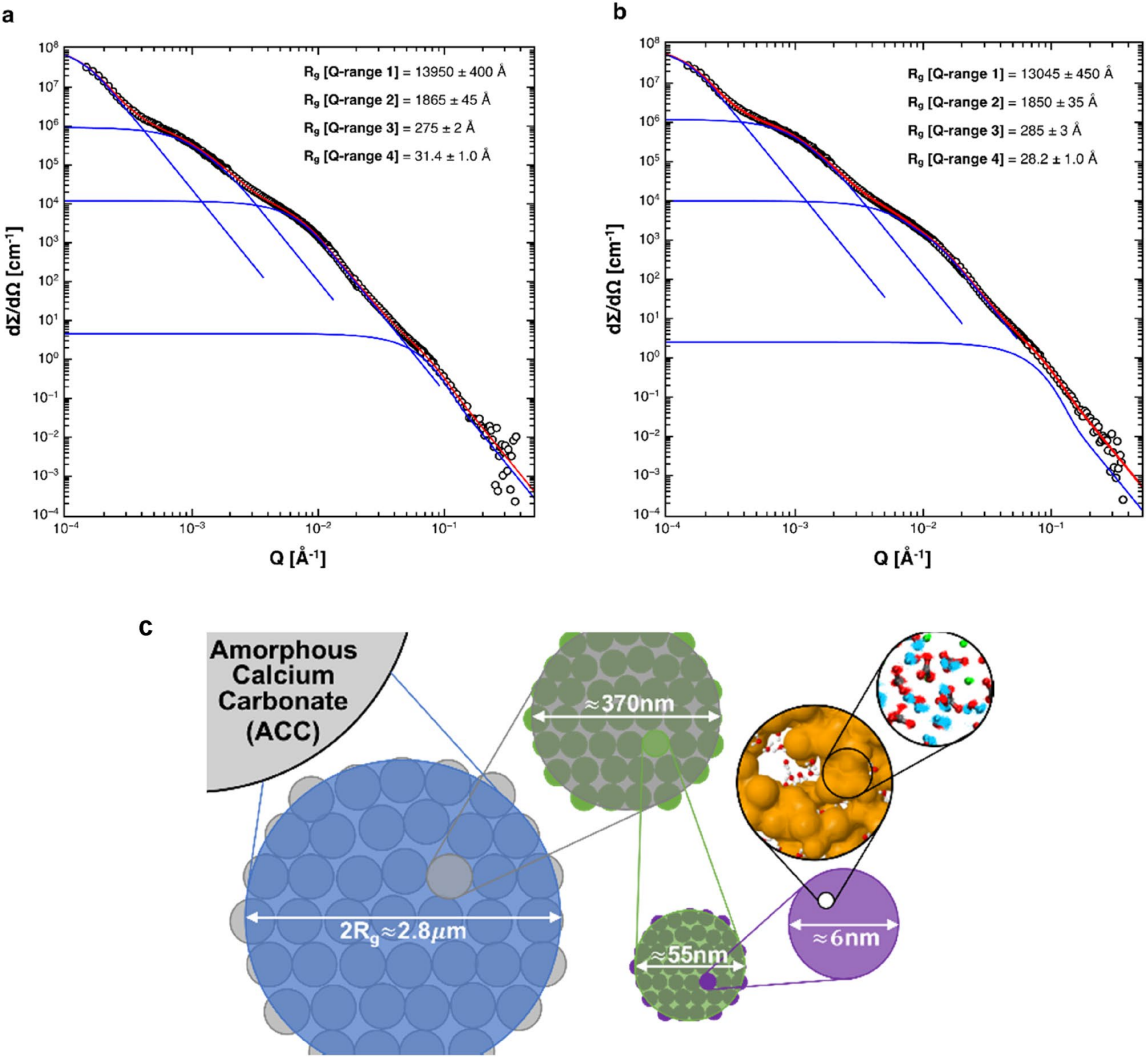
(**a**) Small and ultra-small angle neutron scattering from protonated and deuterated ACC. Neutron scattering cross-sections from: (**a**) protonated and, (**b**) deuterated ACC. The experimental data is given by empty circles. Red lines correspond to the overall fit model of the scattering cross-section based on a four-component Beaucage form factor model; the four individual components are plotted by blue lines. (**c**) Schematic visualization of the hierarchical organization of ACC precipitated at high pH as revealed in this work from a combination of neutron scattering analyses in the wide-, small- and very small-angle regimes.

Small and ultra-small angle neutron scattering measurements allowed us to probe the mesostructural organisation of the ACC precipitate up to length scales of about 3 µm. The collected scattering patterns of deuterated and protonated ACC samples followed a power law Q − p with p = 4, which is characteristic for morphologies with smooth interfaces (Fig. 5a,b). The scattering data showed multiple independent Guinier regimes that are suggestive of a hierarchically-ordered multilevel meso-scale structure formed by smaller particles aggregating to larger particles. A multi-level Beaucage fit^30^ revealed four independent structural levels whose characteristic length scales well align with our previous results. The smallest building unit detected by small-angle neutron scattering features a gyradius of about 3 nm, which is comparable in size to the fundamental nanodomains in our ultrastructural ACC model. These fundamental units build up the second structural level, with a characteristic size of 55 nm that agrees with particle diameters observed of particles in bright field TEM and also corresponds to the size of crystalline domains which developed upon beam exposure in high-resolution TEM analysis (see Fig. 4). The third and fourth levels are in the size range 370 nm and 2.8 µm respectively. When taking these results together, they suggest that ACC formation proceeds by a consecutive multistep aggregation process that starts from distinct fundamental building units in the low-nanometre range (Fig. 5c).

**Figure 6:**
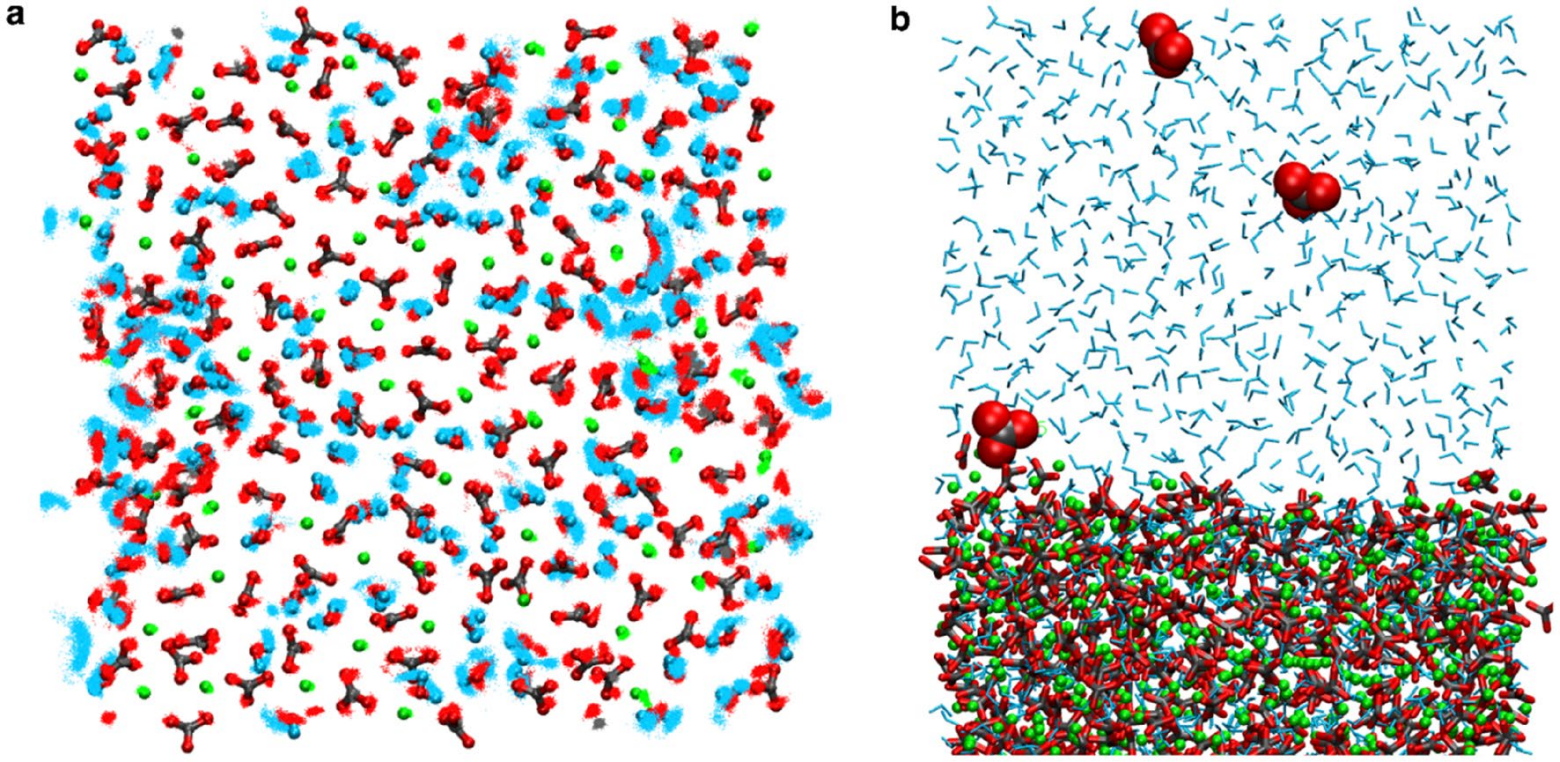
Molecular dynamics simulations on the structural stability of the ultrastructural model (Ca: green, O: red, C: grey, H: blue). (**a**) Cross-section through a cubic, periodic box of the ACC model with an edge length of 42.97 Å containing 1000 CaCO3 and 1200 H2O. The structure is best described in terms of a glass/viscous gel of CaCO3 with occluded water. The light-blue point clouds represent atom positions within a timeframe of 2 ns. Calcium and carbonate ion positions are comparably rigid, while water shows viscous mobility (see Supplementary Fig. S8 for diffusion constants). (**b**) Projection of the same ACC model in contact with a slab of water—the viewing direction is along the ACC surface. Carbonate ions are continuously released and re-adsorbed by the interface leading to an interface in dynamical equilibrium, whereas no release of calcium ions was observed.

We used our nano-domainal structure model as the basis for a series of molecular dynamics simulations off (i) the bulk ACC model and (ii) an interface of ACC and water. Our molecular dynamics studies employed state-of-the-art interaction potentials that have been used in seminal studies^31^ revealing solute clusters formation in calcium carbonate solutions therefore allowing us to discriminate between solute and solid states in calcium carbonate/water systems. Figure 6a highlights the viscous gel-like state, or, glassy nature of the calcium carbonate nano-domains and the embedded hydrate water in ACC. Nearest-neighbour analyses reveal that the hydrate water does not percolate through the structure, but is limited to nanometre-sized domains. In these nano-domains, water molecules and carbonate ions, to a lesser extend, show both orientational and translational flexibility. This flexibility leads to a rather viscous overall mobility via nano-domain deformation and migration in the precipitate with diffusion constants four to five orders of magnitude lower than those observed in aqueous calcium carbonate solutions (Supplementary Fig. S6). These molecular dynamics analyses are consistent with the results from Monte-Carlo simulations and, most significantly, they extend the beforehand static structural information of the ACC model to its nanosecond-scale dynamics. We also explored the structural stability of the ACC model with respect to interfacial water (Fig. 6b). When we exposed our ACC model to a 15 nm-sized film of water, on average five carbonate ions dissociated from the ACC hydrate surface. While a dynamic equilibrium of carbonate dissociation and re-association was observed during 10 ns simulation runs, no calcium release was observed for a surface area of 37 nm^2^. The atomic arrangement and the degree of hydration of the ACC model remained constant upon exposure to water, indicating that our structural model is valid, as it describes a metastable precipitate that does not spontaneously disintegrate when in contact with water.

## Discussion

The methodology presented here allows us to build a detailed picture of the structure and dynamics of seemingly amorphous intermediate phases in mineralization processes. The presence of distinct and repetitive nanodomains in ACC and its multiple levels of hierarchical organization suggest that ACC forms by consecutive steps of particle aggregation, starting from well-defined and nanosized building blocks pre-formed in the mother solution. This is in line with recent studies that have shown the spontaneous formation of multi-centred coordination clusters in calcium carbonate solutions^31,32^. These so-called prenucleation clusters^32^ have been found to be a few nanometres in size (1 to 4 nm—in good agreement with our observations)^26,31–33^ and represent a stable population of solutes which form spontaneously, without having to overcome an energetical barrier^34^. Up to now, their specific role in mineralisation processes has been a matter of debate. Our results support the view that the aggregation of these solute clusters drives the formation of ACC. This hierarchical organization might also explain how polyelectrolytes, such as proteins, can control the formation of the complex structures found in carbonate shells and exoskeletons: by serving as spacers, capping or bridging agents, biopolymers or biomimetic polymers additives may regulate and mediate the individual aggregation steps, intercalating between structural units and/or replacing counterions found within the water-rich channels. The low solubility of calcium as opposed to carbonate revealed by our molecular dynamics simulations would suggest very limited opportunity for a shift in isotopic composition for calcium upon crystallization while this would not be the case for oxygen and point to very limited exchange of magnesium for calcium after ACC formation. Our approach for solving the structure of ACC is equally applicable to other mineral systems with weakly ordered or amorphous intermediates during their formation, be they of biological or synthetic origin. Our findings contribute to a new molecular understanding of mineral formation via amorphous precursors, refining our view on biogenic and abiotic mineral formation^2,35,36^, opening up novel concepts for environmentally sustainable antiscalants^11^ or biomimetic materials^13,37,38^.

## Methods

### Synthesis of ACC

We used a previously reported procedure to synthesise the ACC samples used in this study^19^. Briefly, 100 ml of a freshly prepared 50 mM CaCl_2_ solution (pH 6.6) was placed in a centrifuge bottle. A 12 × 12 cm^2^, 75 μm thick polyethylene sheet was partially pushed into the bottle to form a pouch. A 1 cm diameter (~ 10 g) steel ball was placed into the pouch together with 100 ml of 50 mM Na_2_CO_3_ + 0.5 g of NaOH (pH 13.7). The bottle was capped and placed in an ice bath for an hour before it was placed in a Sorvall RC-5 centrifuge that was cooled to 4 °C, and the sample was spun at 3500 rpm 3 to 5 min. The supernatant liquid was then removed (pH 13.0) and the remaining precipitate washed in a few ml of ethanol. The precipitate was then separated by vacuum filtration (10–15 μm pore size and ~ 0.1 bar pressure) and was subsequently dried for 1–2 h. The same procedure, except for the use of deuterated chemicals (D_2_O and NaOD), was used to make the deuterated sample. The samples were found to contain one water molecule per formula unit. X-ray diffraction data collected from fresh samples demonstrated a lack of sharp peaks characteristic of a crystalline material and the presence of the broad scattering peaks characteristic of an amorphous or nano-crystalline material. These diffraction patterns were found not to change even after samples were stored for many months and even shipped to the synchrotron.

### Neutron scattering

As reported previously^19^, neutron data were collected on the NOMAD instrument at the Oak Ridge National Laboratory Spallation Neutron Source. The sample was loaded into a 5 mm diameter quartz capillary tube inside a glove box under a nitrogen atmosphere, sealed and quickly transported to the instrument. Scattering data were collected in 30 min-frames at room temperature in an Argon atmosphere for a total of 1.5 h. The standard instrument data reduction software was used to normalise the data and subtract the background (Supplementary Fig. S1)^39^.

### X-ray scattering

X-ray scattering data were collected on beamline 11-ID-B at the Advanced Photon Source, Argonne National Laboratory using monochromatic X-rays of energy 58.7 keV (λ = 0.2114 Å). The sample was loaded into a 1 mm diameter polyimide capillary which was sealed with epoxy resin and shipped to the beamline about 48 h before data collection in a temperature-stabilised container. Data were collected at room temperature in transmission using a Perkin Elmer amorphous silicon image plate detector using an exposure time of 30 min. Data from an empty polyimide capillary was also collected. The Fit2D program was used to determine the sample to detector distance and tilt parameters, using a CeO standard, and to integrate the raw two-dimensional data to give standard one-dimension scattering patterns. The total scattering factor S(Q) (Supplementary Fig. S1) was obtained using PDFgetX2 to subtract the empty capillary data and apply the normal corrections^40^.

### EPSR simulations

It is impossible to decompose a total radial distribution function into a set of partial pair distribution functions. Thus, we used a Monte Carlo simulation, constrained by our neutron and X-ray scattering data, to determine a set of atomic coordinates from which we then calculated the partial pair distribution functions (pPDF). This simulation was carried out using the Empirical Potential Structure Refinement method (EPSR)^41^. The program searches for distributions of atoms that are consistent with the experimental data and sets of constraints such as interatomic potentials, effective charges on the atoms, assumed molecular shapes and assumed minimum atom–atom distances through a series of simulation loops that accept or reject atom moves based on the Metropolis algorithm. The interatomic potentials consisted of sums of Lennard–Jones potentials, to represent the short-range repulsive forces, and pseudo-Coulomb potentials, to represent the attractive long-range forces (Supplementary Information 1). The EPSR program uses sums of these pairwise atomic potentials to give a Reference Potential which is used to equilibrate the simulations. Differences between the experimental data and data calculated from the atomic positions in the simulation box are used to give an Empirical Potential. This is added to the Reference Potential after the initial equilibration, and the simulation is continued moving atoms and periodically updating the Empirical Potential until convergence is achieved. A simulation box containing 1000 Ca atoms, 1000 CO_3_ molecules, and 1000 H_2_O molecules was created, with an atomic number density of ρ = 0.1 Å^−3^. The resulting simulation was stopped after reaching convergence after 6885 iterations.

### Model building

The sharp peaks observed in the partial pair distribution function (pPDF) for Ca–Ca (Supplementary Fig. S2) at about 4, 6 and 8 Å and the associated sharp peaks observed in the Ca–Ca–Ca bond angle distribution function (Supplementary Fig. S3) at about 55° and 90° were used to define the basic lattice with Ca1 being 4 Å to Ca2 and Ca5, Ca1 being 8 Å to Ca4, Ca1 being 6 Å to Ca6, Ca2 being 4 Å to Ca3, the Ca5–Ca1–Ca2 angle being 55° and the Ca3–Ca2–Ca1 angle being 90° (Supplementary Fig. S5). Now the carbon groups can only possibly be located between calcium atoms along the a direction in order to approximately satisfy the doublet centred close to the 3 Å Ca–C distance in the Ca–C pPDF given the need to satisfy the Ca–O distance of 2.4 Å in the Ca–O pPDF and the intermolecular C–O distance of 3.8 Å in the C–O pPDF. The doublet in the Ca–C pPDF at about 3 Å is explained by the carbonate C atoms being in two positions giving Ca1–C1 and Ca6–C2 distances of 2.8 Å and 3.3 Å to match the doublet peak positions. The orientation of the carbonate groups is further constrained by the O–O distances in the O–O pPDF forcing them to be arranged in the same orientation along the c-axis although they can have any orientation along the a and b axes. About 70% of carbonate groups have monodentate coordination with calcium and 30% have bidentate coordination with calcium. Two water molecule positions can be identified: one on the [110] crystal face and the other on the [011] crystal face giving rise to the 2.5 Å peak in the Ca–Ow pPDF and the 3.2 Å and 3.6 Å peaks in the C–Ow pPDF. The oxygens are arranged with the lone pairs pointing towards two Ca atoms and the hydrogens arranged away from the crystal faces which gives rise to the 3.1 Å peak in the Ca–H and 3 and 4 Å peaks in the C–H pPDFs. The peaks at 2.7 Å in the Ow–Ow pPDF and the peaks between 2 and 3.4 in the Ow–H pPDF are due to water-water correlations in the water channels. The 1.8 peak in the O–H pPDF corresponds to a channel water-carbonate group interaction on the [101] plane orientated with the hydrogen pointing towards the crystal face. The atomic coordinates of this average structure are contained in (Supplementary Table S1). The peaks in the pair distribution functions and bond angle distribution functions are wide demonstrating a degree of disorder in the atomic structure which may be due to the small size of the ACC particles and the fact that some calcium atoms are hydrated while others are not.

### Transmission electron microscopy (TEM)

High-resolution (HR) TEM and selected area electron diffraction (SAED) were performed with a double aberration-corrected FEI Titan Themis 60–300 TEM operated at 300 kV. The electron beam irradiation of the imaged area was minimized to avoid beam damage to the sample (beam current around 140 pA). The samples were synthesized as given above, dried over silica for 3 days, and eventually annealed for 2.5 h at 200 °C. The dry powder was suspended in water-free ethanol using ultrasound and was drop cast onto Lacey carbon TEM sample grids (400 mesh). The coherence lengths L_hkl_ of (crypto-)crystalline regions were determined by applying the Scherrer equation^42^, L_hkl_ = (k λ)/(β cosθ), (where L_hkl_ is the mean crystallite size, k is a dimensionless shape factor (here: 0.89 for spherical shape), λ is the wavelength of the electrons λ = 1.97 pm with an energy of 300 keV, β is the line broadening (integral width), and θ is the Bragg angle) to rotationally averaged SAED ring patterns assuming Gaussian peak shapes giving estimated particle size of about 1.8 nm. The observed lattice spacing of 0.24076 nm could also be assigned to CaO (d_200_ = 0.24076 nm, ICSD no. 51409), which may form when CaCO_3_ is exposed to ultra-high vacuum and radiation for an extended period^43,44^. It may also be assigned to the first signal of pristine ACC when taking into account that dehydration may lead to distinct lattice distortions. The lattice spacings of calcite (ICSD no. 18164) and vaterite (AMCSD 0019138) were retrieved from pertinent crystallographic databases.

### Small and ultra-small angle neutron scattering (SANS, VSANS)

Small-Angle Neutron Scattering (SANS) experiments were performed on KWS-2 and KWS-3 instruments operated by Jülich Centre for Neutron Science (JCNS) at the Heinz Maier-Leibnitz Zentrum (MLZ) in Garching, Germany^45,46^. Focusing SANS instrument KWS-3 covered a broad *Q*-range from 0.0001 to 0.35 Å^−1^ at sample-to-detector distances of 9.2, 1.25, 0.25 and 0.05 m. This resolution was reached by a toroidal mirror with focus-to-focus distance 22 m, entrance aperture 2 × 2 mm^2^, wavelength *λ* = 12.8 Å (Δ*λ/λ* = 17), and two-dimensional position-sensitive scintillation detector with diameter 9 cm and pixel size 0.32 mm. To improve scattering statistics above Q = 0.02 Å^−1^ experiments were also carried out at the classical pinhole SANS instrument KWS-2 at sample-to-detector distances of 2 and 8 m (corresponding collimation length of 4 and 8 m), and wavelength of *λ* = 5 Å (Δ*λ/λ* = 10%). Within the configurations mentioned above, KWS-2 covers a *Q*-range from 0.01 to 0.45 Å^−1^. ACC(d) and ACC(h) powders were placed to a demountable quartz cell with a path length of 0.1 mm. The data reduction, analysis, background subtraction, and fitting were performed using the software QtiKWS (V. Pipich, 2019, http://www.qtikws.de). A multi-level Beaucage fit was applied^30^. Thus, the combination of the two SANS instruments allowed us to investigate structural sizes from 6 Å to 3 µm. The scattering curves obtained were analyzed with

$$\frac{d\Sigma }{{d\Omega }}(Q) = \sum\limits_{i = 1}^{m} {\left( {G_{i} \exp \left( { - \frac{{Q^{2} R_{gi}^{2} }}{3}} \right) + B_{i} \exp \left( { - \frac{{Q^{2} R_{{g\left( {i + 1} \right)}}^{2} }}{3}} \right)\left\{ {\frac{{erf\left( {\left[ {QR_{gi} /\sqrt 6 } \right]^{3} } \right)}}{Q}} \right\}^{pi} } \right)} ,$$

where *m* is the number of the structural levels (in our case *m* = 4), *G*_*i*_ forward scattering and *B*_*i*_ power low amplitude of ith structural level with the fixed exponent *p*_*i*_ = 4.

### Molecular dynamics

Simulations were carried out using the LAMMPS package using a time step of 1 fs. For the sake of best comparability to DOLLOP solutions by Gale et al., the interaction potentials were fully adopted from these studies (Supplementary Information 2)^31,34,47,48^. For the force evaluations, a cutoff distance of 11 Å was applied, combined with the damped shifted force approach to the Coulomb interactions using a damping factor of α = 0.05 Å^−1^^48^. Each of the simulation models were first relaxed at 300 K and 1 atm within 1 ns runs. For the data production, molecular dynamics runs at ambient conditions of 5 and 10 ns were performed for the bulk ACC system and the interface model, respectively.

## Supplementary Information

Supplementary Information.
