# Supplementary material for: The nano- and meso-scale structure of amorphous calcium carbonate
Source: Sci Rep. 2022 Apr 27;12:6870. doi: 10.1038/s41598-022-10627-9 (PMC9046151; doi:10.1038/s41598-022-10627-9)
Supplement: Supplementary file 1 — Supplementary Information. [file 41598_2022_10627_MOESM1_ESM.docx]

**The nano- and meso-scale structure of amorphous calcium carbonate**

***— Supplementary Information —***

*Simon M. Clark,^*,1,2^ Bruno Colas,^1,2^ Dorrit E. Jacob,^3^ Joerg C. Neuefeind,^4^ Hsiu-Wen Wang,^4^ Katherine L. Page,^4^ Alan K. Soper,^5^ Philipp I. Schodder,^6^ Patrick Duchstein,^7^ Benjamin Apeleo Zubiri,^8^ Tadahiro Yokosawa,^8^ Vitaliy Pipich,^9^ Dirk Zahn,^7^ Erdmann Spiecker,^8^ and Stephan E. Wolf.^*,6,10^*

1. School of Engineering, Macquarie University, Macquarie Park, NSW 2113, Australia.
2. Australian Centre for Neutron Scattering, Australian Nuclear Science and Technology Organisation, Locked Bag 2001, Kirrawee DC, NSW 2232, Australia.
3. Research School of Earth Sciences, The Australian National University, Canberra, ACT 2600, Australia.
4. Spallation Neutron Source, Oak Ridge National Laboratory, Pak Ridge, TN 37831, USA.
5. ISIS Facility, Rutherford Appleton Laboratory, Chilton, Didcot, Oxon, OX11 0QX, UK.
6. Department of Materials Science and Engineering (WW), Institute of Glass and Ceramics (WW3), Friedrich-Alexander-University Erlangen-Nuremberg (FAU), Martensstrasse 5, 91058 Erlangen, Germany.
7. Department of Chemistry and Pharmacy, Chair for Theoretical Chemistry / Computer Chemistry Centre (CCC), Friedrich-Alexander-University Erlangen-Nuremberg (FAU), Nägelsbachstrasse 25, 91058 Erlangen, Germany.
8. Institute of Micro- and Nanostructure Research (IMN) & Center for Nanoanalysis and Electron Microscopy (CENEM), Interdisciplinary Center for Nanostructured Films (IZNF), Friedrich-Alexander-University Erlangen-Nuremberg (FAU), Cauerstraße 3, 91058 Erlangen, Germany.
9. Jülich Centre for Neutron Science (JCNS), Forschungszentrum Jülich GmbH, Outstation at FRM II, Lichtenbergstrasse 1, D-85747 Garching, Germany
10. Interdisciplinary Center for Functional Particle Systems (FPS), Friedrich-Alexander University Erlangen-Nürnberg (FAU), Haberstrasse 9a, 91058 Erlangen, Germany

* To whom correspondence should be addressed: [simon.clark@mq.edu.au](mailto:simon.clark@mq.edu.au) & [stephan.e.wolf@fau.de](mailto:stephan.e.wolf@fau.de)

**Supplementary Figure S1. Normalised and corrected scattering intensity S(Q) of Amorphous Calcium Carbonate** produced using (a) neutrons and (b) x-rays as a function of scattering vector Q (Q=4πsinθ/λ, where θ is half of the scattering angle and λ is the wavelength). The blue markers show the experimental data and the yellow line is the fit to the Monte Carlo model.

**
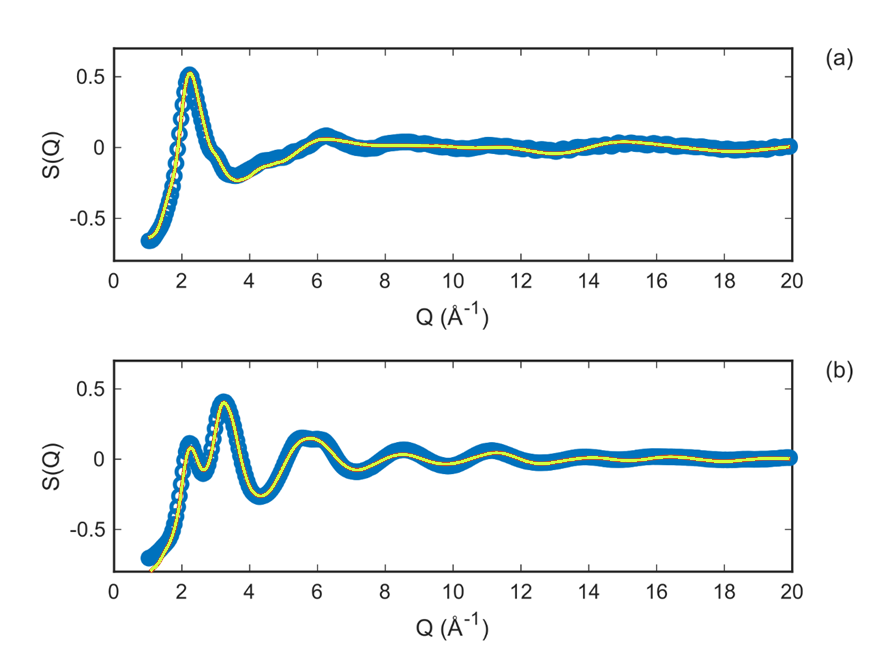
**

**Supplementary Figure S2.** **Partial pair distribution functions determined for each atom-atom pair** in the Monte-Carlo simulation. The partial pair distribution function is the probability of finding an atom of type Y from an atom of type X as a function of distance from atom of type X. Intense sharp peaks in these functions demonstrate the localisation of atoms at particular pair wise distances.

**
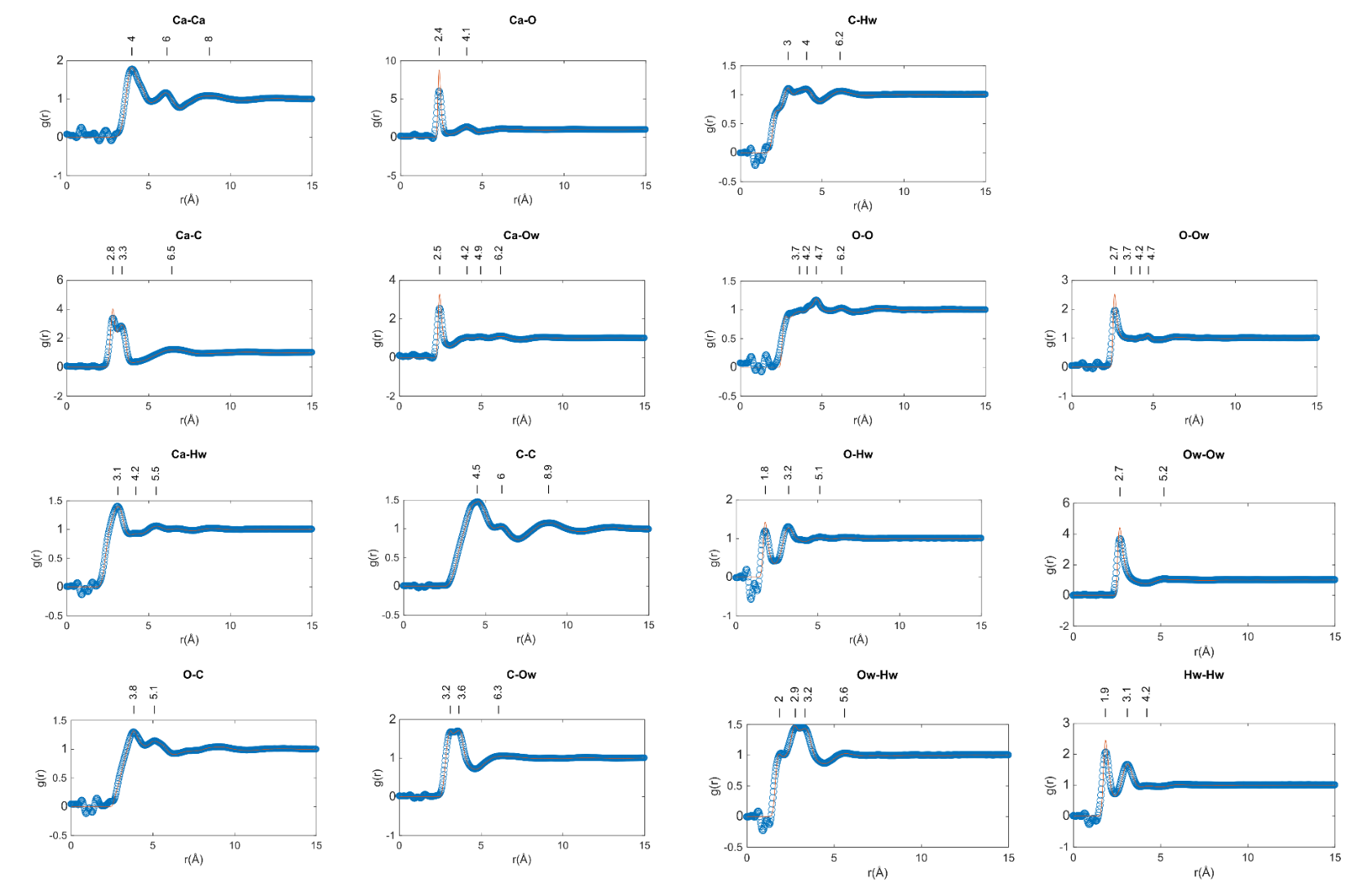
**

**Supplementary Figure S2 (continued)**

**
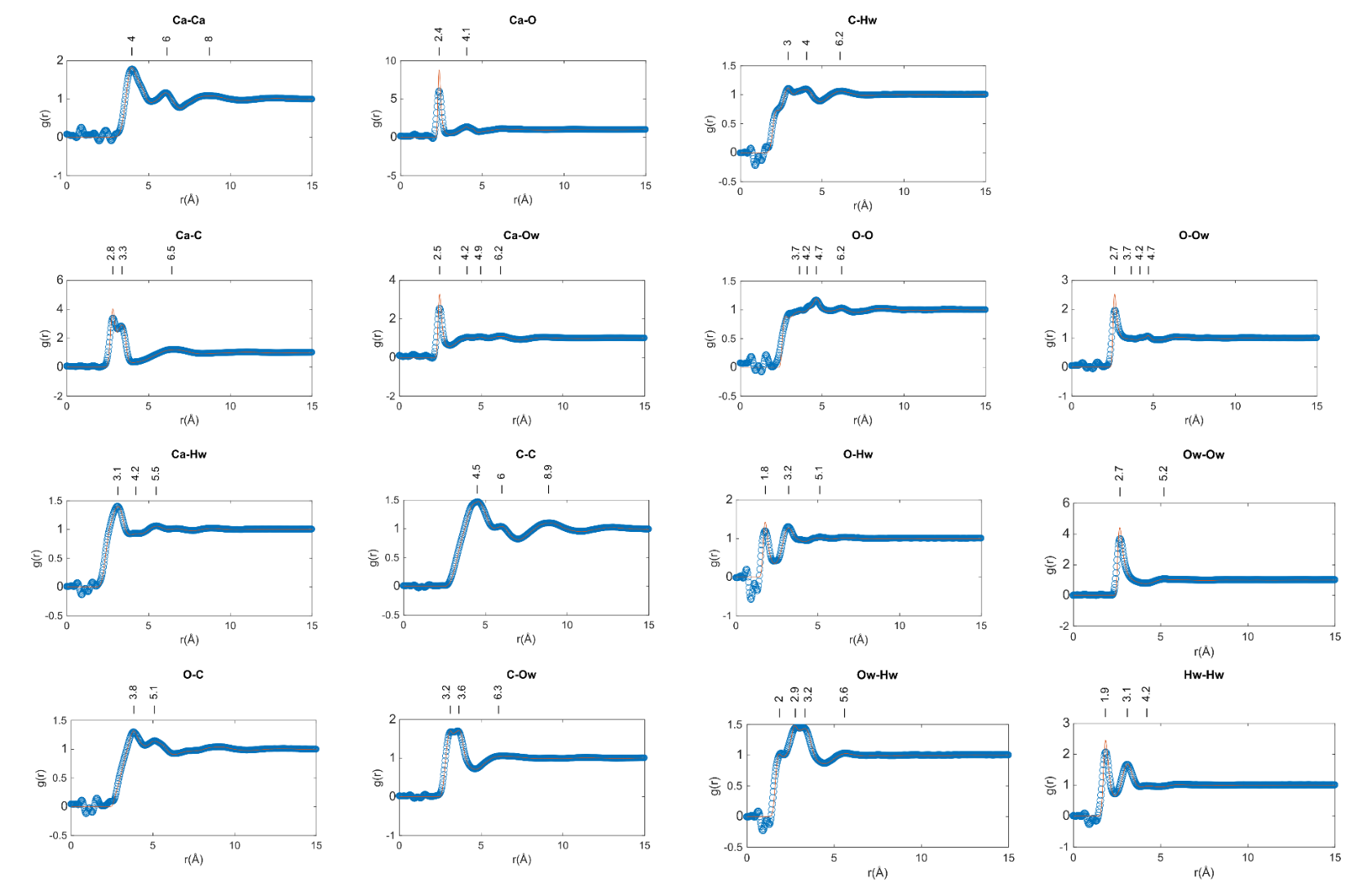

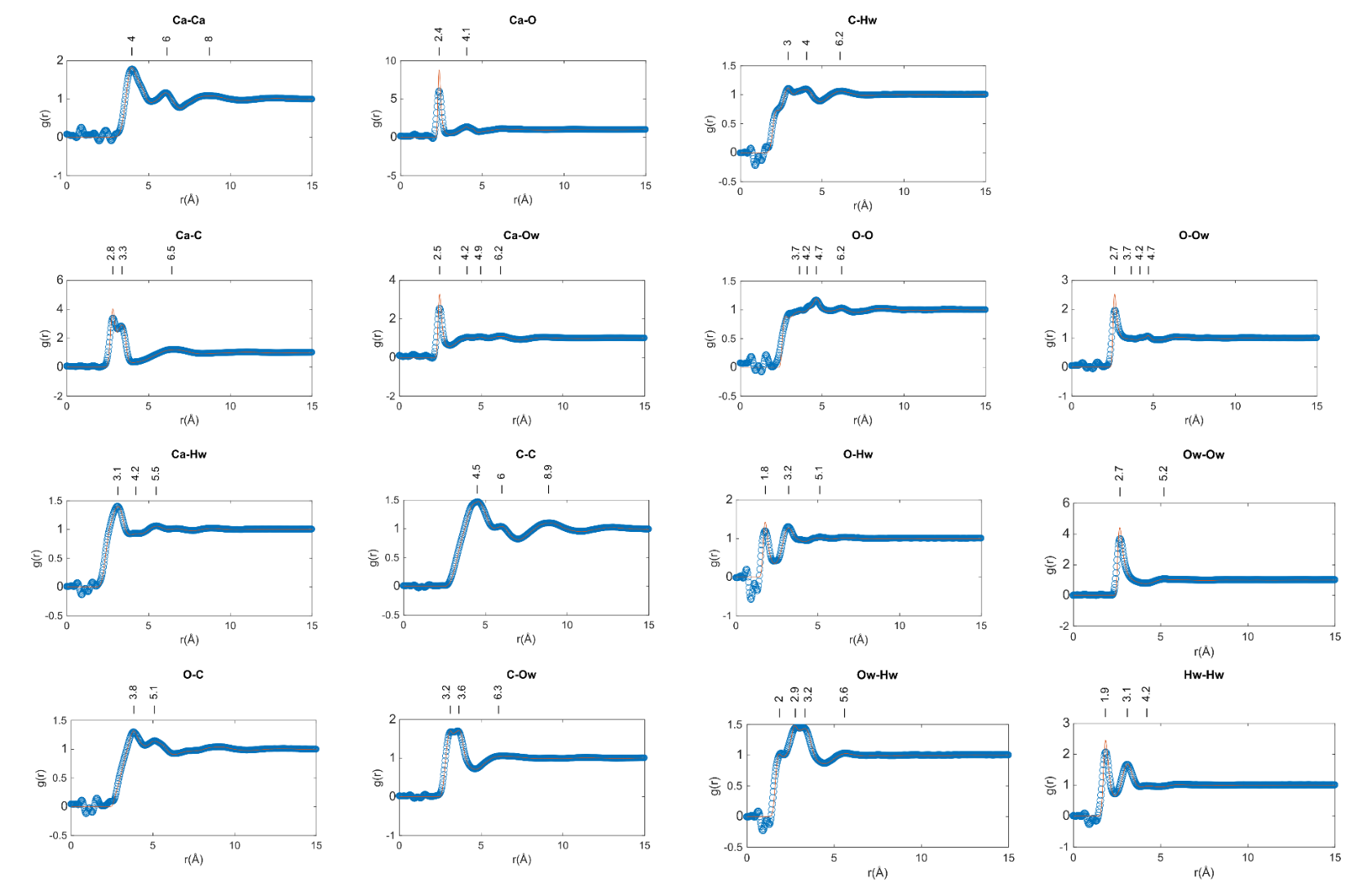
**
**
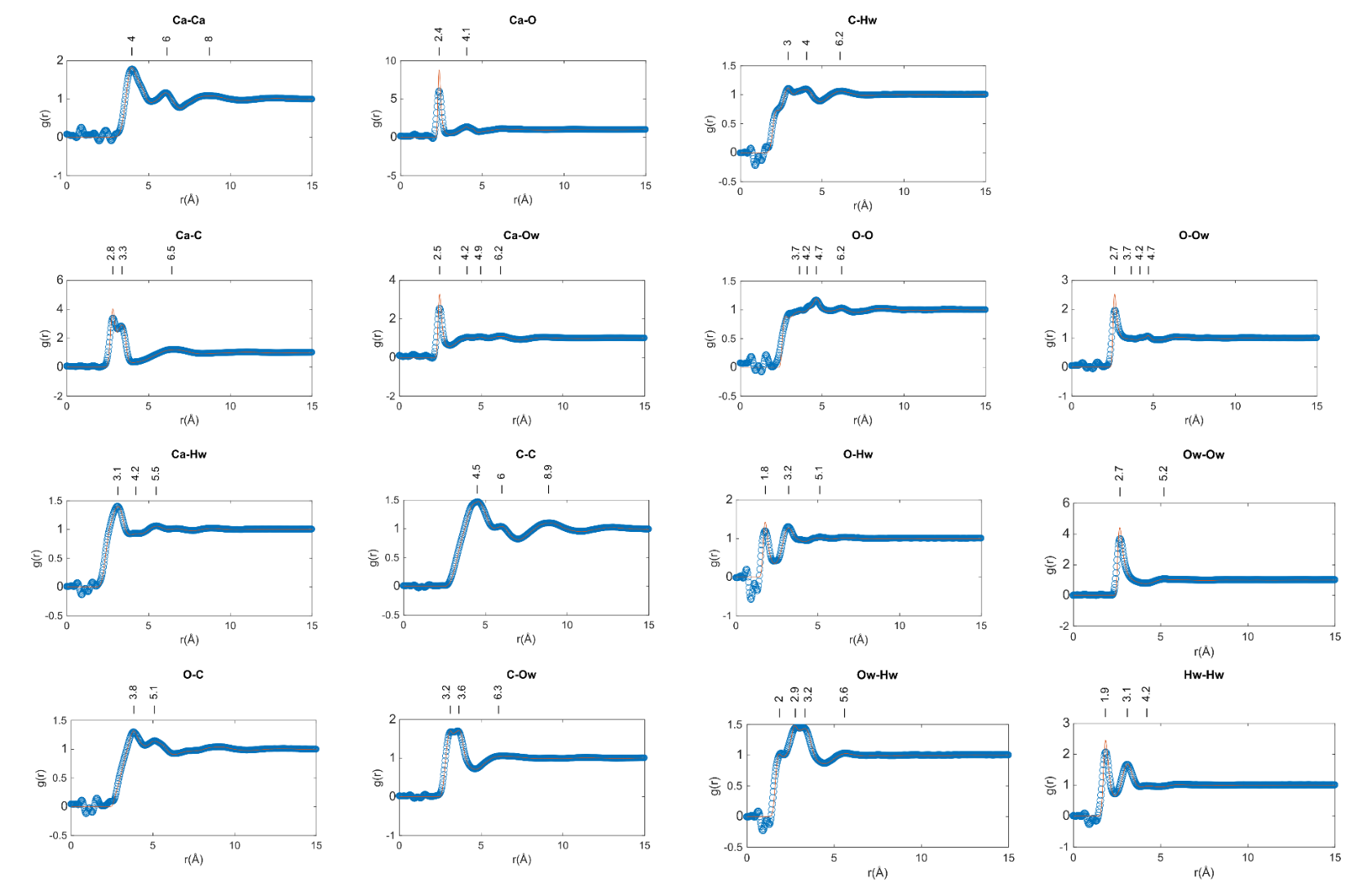
**

**Supplementary Figure S3. Bond angle probability distributions** for selected X-Y-Z atom types obtained from the Monte-Carlo simulation. These angles were calculated using cut off distances to ensure that only first nearest neighbour atoms were included in the calculation. The sharp peaks in the distributions demonstrate a fairly rigid atomic structure.


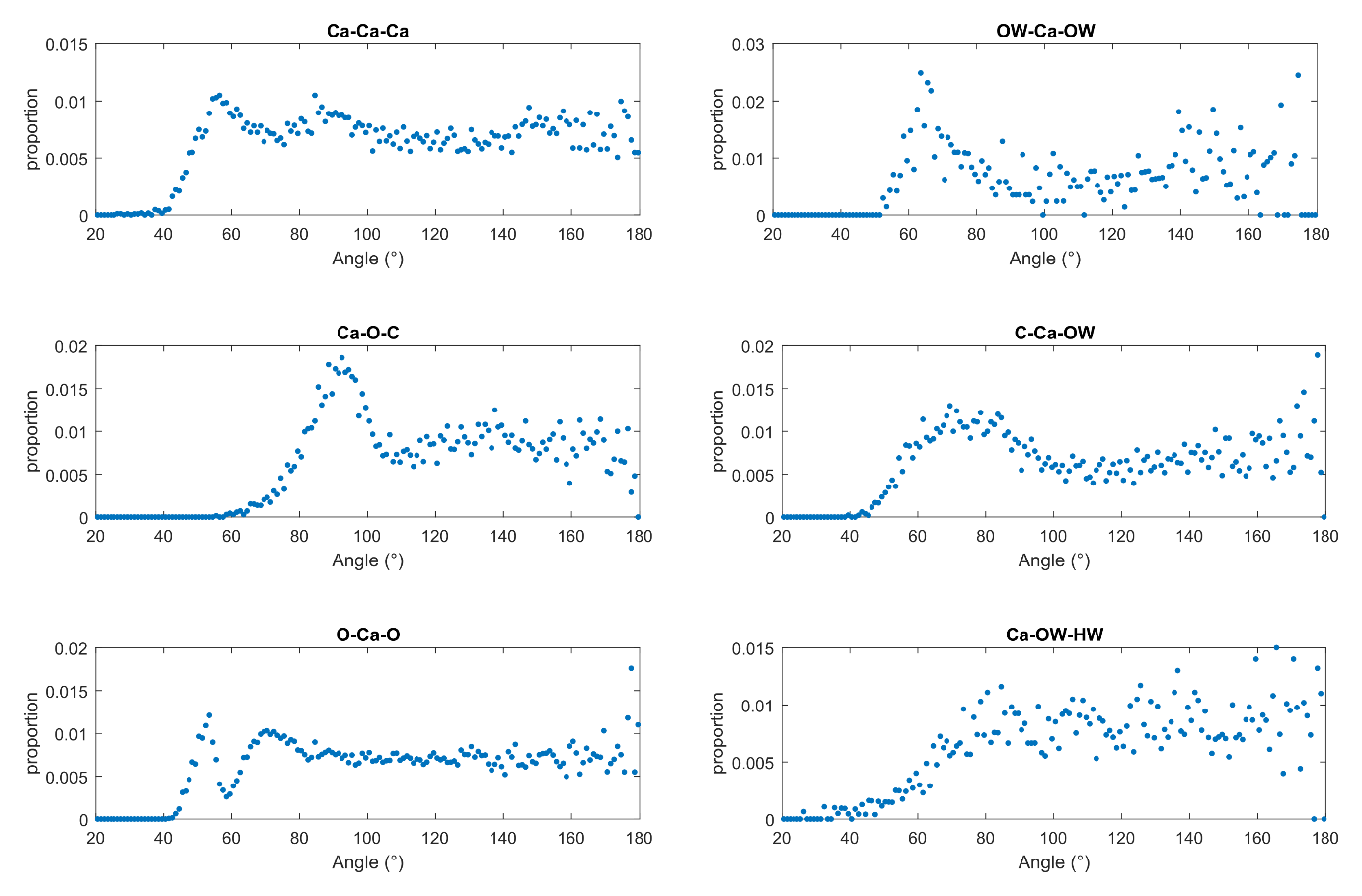


**Supplementary Figure S4. The average atomic structure of Amorphous Calcium Carbonate.** Projections of the average atomic structure of Amorphous Calcium Carbonate onto (a) the ac plane and (b) the ab plane. Calcium atoms are shown as green spheres, carbon atoms as grey spheres, oxygen atoms as red spheres and hydrogen atoms as white spheres. This structure was determined using model building to find the best match the distances and bond angle distribution functions derived from the coordinates produced by the Monte-Carlo simulations.

**
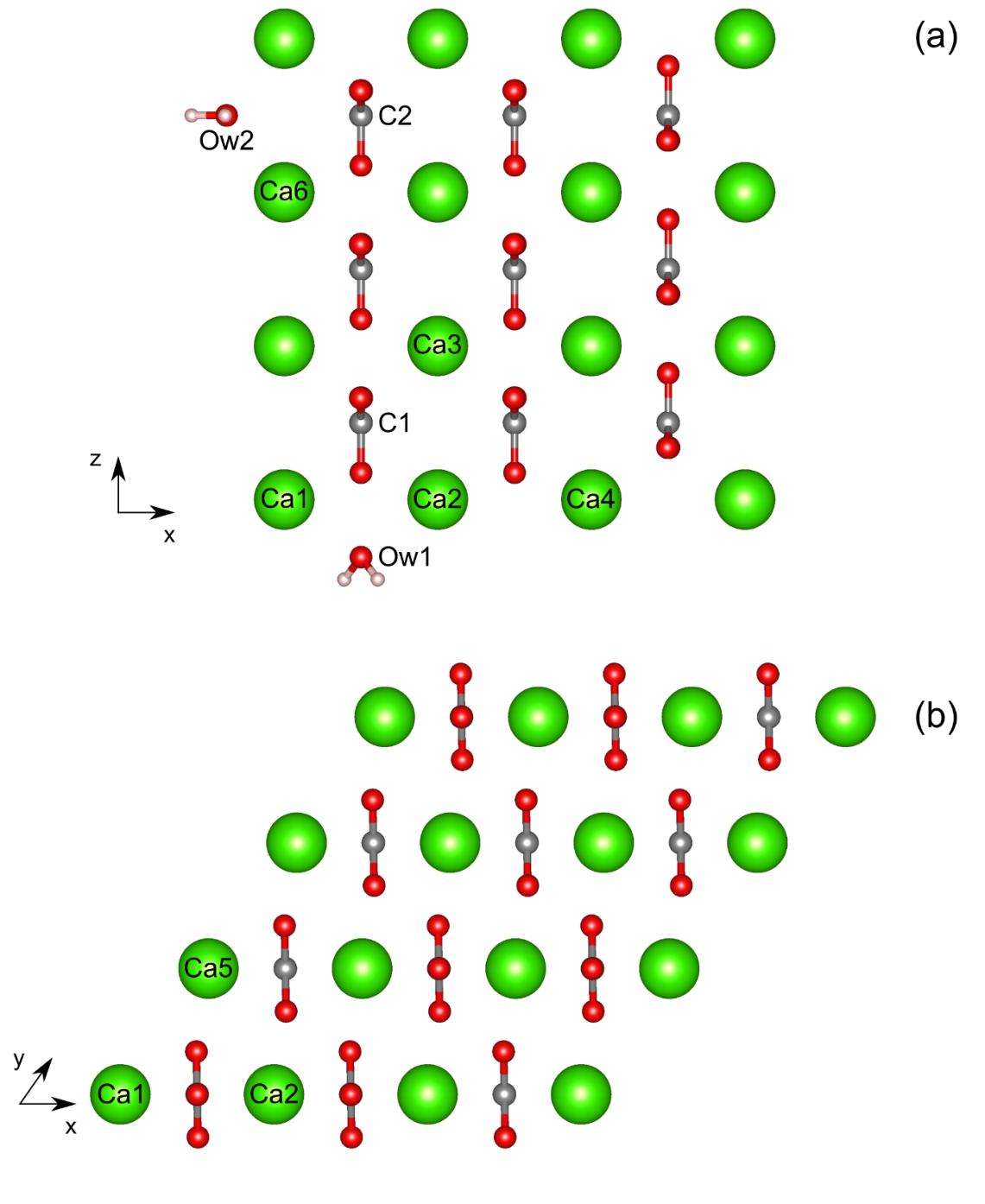
**

**Supplementary Table 1. Lattice parameters and fractional atomic coordinates for the average amorphous calcium carbonate atomic structure.** The coordinates for the two carbonate orientations are representative. The orientations of the carbonate groups are constrained to be the same along the c-axis while they can be the same or different along the a and b axes.

|  | **a = 4 Å** | **b = 4 Å** | **c = 4 Å** |
| --- | --- | --- | --- |
|  | **α = 90^o^** | **β = 90^o^** | **γ = 55^o^** |
|  |  |  |  |
|  | **x** | **y** | **z** |
| **Ca1** | 0 | 0 | 0 |
| **C1** | 0.5 | 0 | 0.5 |
| **O1a** | 0.5 | 0 | 0.18 |
| **O1b** | 0.5 | 0.28 | 0.66 |
| **O1c** | 0.5 | -0.28 | 0.66 |
| **C2** | 0.5 | -0.22 | 2.5 |
| **O2a** | 0.5 | -0.22 | 2.18 |
| **O2b** | 0.5 | 0.06 | 2.66 |
| **O2c** | 0.5 | -0.5 | 2.66 |
| **Ow1** | 0.5 | 0 | -0.375 |
| **H1a** | 0.5 | 0.19 | -0.52 |
| **H1b** | 0.5 | -0.19 | -0.52 |
| **Ow2** | -0.375 | 0.2 | 2.5 |
| **H2a** | 0.55 | 0.29 | 2.5 |
| **H2b** | 0.55 | -0.09 | 2.5 |

**Supplementary Table 2. Positions of peaks in the x-ray and neutron scattering data.** Data from this work, Goodwin et al.^1^, Cobourne et al.^2^, and Jensen et al.^3^

| **Peak** | **Radiation** | **Peak position (Å^-1^)** | | | |
| --- | --- | --- | --- | --- | --- |
|  |  | *This Work* | *Cobourne et al.* | *Jensen et al.* | *Goodwin et al.* |
| **1** | X-ray | 2.22 | 2.20 | 2.18 | 2.22 |
|  | Neutron | 2.22 | 1.84 | 1.99 |  |
| **2** | X-ray | 3.14 | 3.15 | 3.33 | 3.33 |
|  | Neutron | 3.2 | 3.15 | 2.98 |  |
| **3** | X-ray | 5.36 | 5.62 | 5.34 | 5.57 |
| **4** | X-ray | 6.23 | 6.19 | 6.31 | 6.24 |
|  | Neutron | 6.23 | 6.19 | 6.19 |  |

**Supplementary Table 3. Correlation distances estimated for maxima in the Total Pair Distribution Functions (tPDFs).** From this work, Goodwin et al.^1^, Cobourne et al.^2^ and Jensen et al.^3^ The studies of Fernandez-Martinez et al.^4^ and Goodwin et al.^1^ only show a portion of the tPDF. Both contain the peaks at 4 Å and 6 Å.

| **Designation** | **Correlation distance (Å)** | | | | | | |
| --- | --- | --- | --- | --- | --- | --- | --- |
|  | *This work* | *Cobourne et al.* | | *Jensen et al.* | | *Goodwin et al.* |  |
|  | Neutron &  X-ray | X-ray | Neutron | X-ray | Neutron | X-ray |  |
| **O-H(D)** | 0.97 | 0.89 | 0.91 |  |  |  |  |
| **C-O** | 1.27 | 1.24 | 1.24 | 1.26 | 1.29 | 1.29 |  |
| **D-D** | 1.6 |  | 1.54 |  |  |  |  |
| **Mg-O** |  | 2.25 | 2.22 |  | 2.23 |  |  |
| **Ca-O** | 2.4 |  | 2.42 | 2.38 | 2.43 | 2.37 |  |
| **Ca-C, C-O, C-Ow** | 3.09 |  | 2.95 | 2.86 | 2.93 | 2.87 |  |
|  | 3.31 |  | 3.32 |  | 3.32 |  |  |
| **Ca-Ca** | 4.07 | 3.85 | 4.08 |  | 4.08 | 4.09 |  |

**Supplementary Table 4. Correlation distances estimated for maxima in the Partial Total Pair Distribution Functions (pPDFs).** From this work, Goodwin et al.^1^, Cobourne et al.^2^, and Jensen et al.^3^

| **Atom pairs** | **Position of maxima in pPDFs (Å)** | | | |
| --- | --- | --- | --- | --- |
|  | *This work* | *Cobourne et al.* | *Jensen et al.* | *Goodwin et al.* |
| **Ca-Ca** | 4 | 3.30 | 3.95 | 3.99 |
|  | 6 | 6.17 | 6.22 | 6.20 |
| **Ca-O** | 2.4 | 2.39 | 2.35 |  |
|  | 4.1 | 4.19 | 4.2 | 4.18 |
| **C-O** | 3.8 | 2.39 | 4.02 | 3.55 |
|  | 5.1 | 4.19 | 5.25 | 5.17 |
| **Ca-C** | 2.8 |  | 2.93 |  |
|  | 3.3 | 3.29 | 3.34 | 3.47 |
|  | 6.5 | 6.12 | 6.68 |  |
| **C-C** | 4.5 | 3.48 | 4.31 |  |
|  | 6 |  | 6.24 |  |
|  | 8.9 |  | 8.73 |  |
| **Ca-Ow** | 2.5 | 2.39 | 2.41 |  |
|  | 4.2 | 4.19 | 4.1 |  |
|  | 4.9 |  | 5.01 |  |
|  | 6.2 | 6.95 | 6.43 |  |
| **O-Ow** | 2.7 |  | 2.77 |  |
|  | 4.7 |  | 4.61 |  |
| **Ow-Ow** | 2.7 |  | 2.7 |  |
|  | 5.2 |  | 5.23 |  |
| **Ca-H(D)** | 3.1 | 2.53 | 2.88 |  |
|  | 4.2 |  | 4.7 |  |
|  | 5.5 |  | 5.72 |  |
| **O-H(D)** | 1.8 | 1.16 | 1.62 |  |
|  | 3.2 | 2.65 | 2.84 |  |
|  |  |  | 3.62 |  |
|  | 5.1 |  | 5.10 |  |
| **H-H (D-D)** | 1.9 | 1.83 | 2.25 |  |
|  | 3.1 |  | 3.16 |  |
|  | 4.2 |  | 5.86 |  |

**Supplementary Figure S5. Comparison of our ACC structure with known calcium carbonate structures**. Representative diagrammatic slices from the atomic structures of known calcium carbonates, equivalent to the ac plane in our nano-ACC structure, selected to illustrate the relative carbonate group orientations are shown. Calcium atoms are represented by filled green circles, oxygen atoms by filled red circles, carbon atoms by filled black circles and hydrogen atoms by unfilled black circles. Atoms not in the selected slice but necessary to fully illustrate the structure are represented by unfilled circles or filled circles with 50% transparency applied. The structures can be seen to divide into two groups either with the carbonate groups in the ab plane or with the carbonate groups perpendicular to the ab plane. Vaterite can been then viewed as an intermediate on the path to either calcite or aragonite and ikaite can be viewed as a low temperature version where the original ACC structure has been “frozen” in.****

**Supplementary Figure S6. Mean square deviation of atomic position as a function of time in molecular dynamics simulation** of bulk ACC models. The diffusion coefficient of water is about one magnitude larger than that of Ca and CO_3_. The offsets
(O_w_ 0.76 Å^2^, H_w_: 1.84 Å^2^, Ca: 0.22 Å^2^, C_c_: 0.21 Å^2^, O_c_: 0.46 Å^2^) denote local/rotational/vibrational degrees of freedom which are substantially larger for water than for calcium and carbonate.


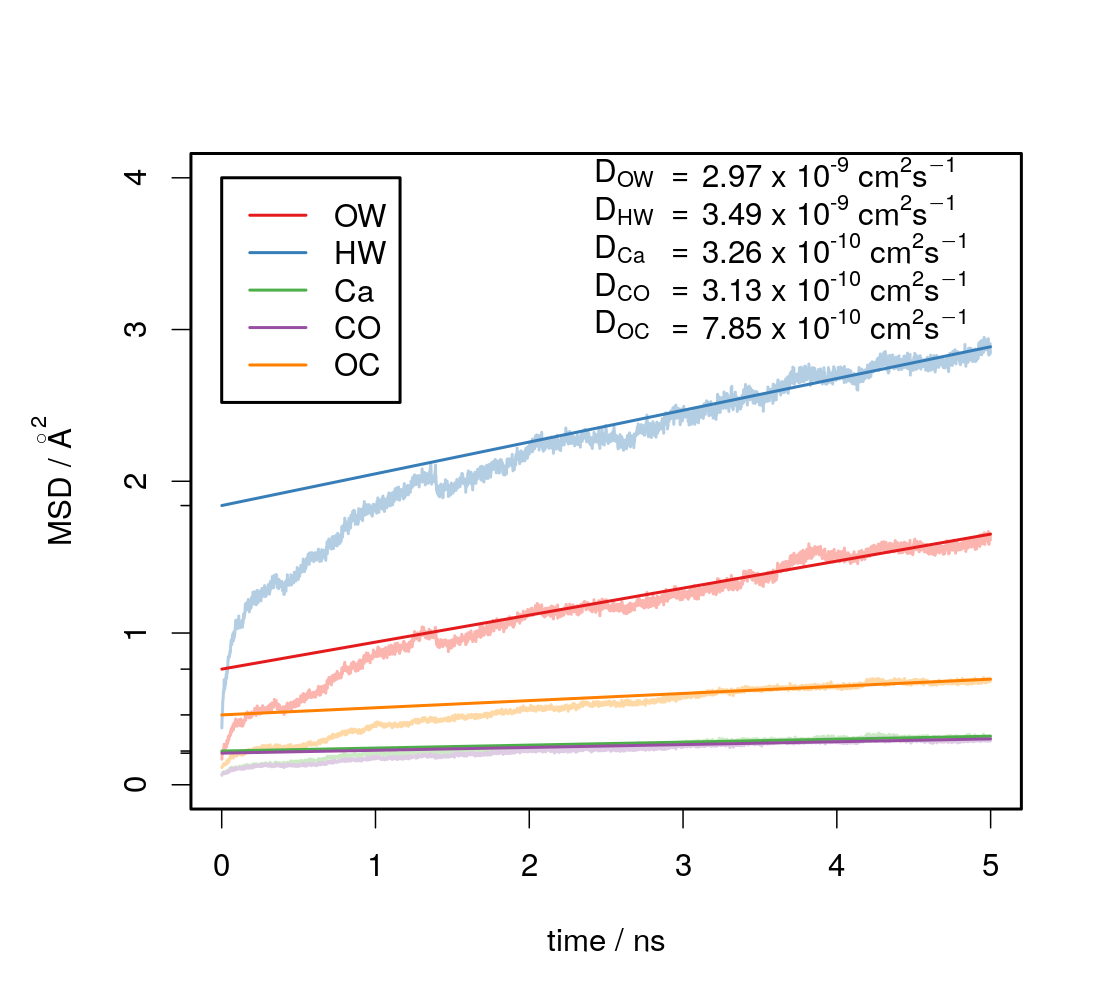


**Supplementary Information 1. Parameters used in the EPSR simulations**^5^.

The atoms involved in the simulation are:

| Ca | calcium |
| --- | --- |
| C | carbon in carbonate |
| O | oxygen in carbonate |
| Ow | oxygen in water |
| H | hydrogen in water |

C and O were set as CO_3_ molecules and Ow and H were set as H_2_O molecules. Each atom or molecular entity (Ca, CO_3_ and H_2_O) was created in an EPSR *mol* file, that includes the molecular geometry (bond length, bond angle), when appropriate, and the density and Lennard-Jones parameters. The parameters used in the EPSR *mol* files are:

| **Atomic number density** | | | **Lennard-Jones parameters** | | | |
| --- | --- | --- | --- | --- | --- | --- |
|  | | ***ρ (Å^-3^)*** |  | | ***ε (kJ.mol^-1^)*** | ***σ (Å)*** |
| Ca | | 0.0134 |  | | 0.26 | 3.1 |
| CO_3_ | | 0.0134 | C | | 0.2 | 3.6 |
|  |  |  | O | | 0.65 | 3.15 |
| H_2_O | | 0.0134 | Ow | | 0.65 | 3.166 |
|  |  |  | H | | 0 | 0 |

The Lennard-Jones parameters ε_αβ_ and σ_αβ_, for pairs of atom types α and β, are determined from these individual atom parameters, ε_α_, ε_β_, σ_α_ and σ_β_, using the Lorentz-Berthelot mixing rule:

ε_αβ_ = (ε_α_ ε_β_)^1/2^

σ_αβ_ = 1/2 (σ_α_ + σ_β_)

**Supplementary Information 2**. **Force fields parameters used in the molecular dynamics simulations.** The applied force field parameters comply with those introduced by Raiteri et al.^6^ and Wu et al.^7^

$U_{buck}(r)=Ae^{-r/\rho}-\frac{C}{r^{6}}$ $U_{LJ}(r)=4\varepsilon\left[\left(\frac{\sigma}{r}\right)^{12}-\left(\frac{\sigma}{r}\right)^{6}\right]$

$U_{bond}(r)=\frac{k}{2}(r-r_{0})^{2}$ $U_{angle}(\theta)=\frac{k}{2}(\theta-\theta_{0})^{2}$

$$U_{improper}(d)=k_{2}d^{2}+k_{4}d^{4}$$

Where the parameters used in the Intermolecular Buckingham Potentials are:

.

| **Buckingham** | ***A* (eV)** | **ρ (Å)** | ***C* (eVÅ^6^)** |
| --- | --- | --- | --- |
| Ca−O | 3161.63 | 0.27151 | 0.000 |
| Ca−C | 120000000.00 | 0.12000 | 0.000 |
| O−O | 63840.20 | 0.19891 | 27.899 |
| O−O_w_ | 12534.46 | 0.20200 | 12.090 |
| O−H_w_ | 396.30 | 0.21700 | 0.000 |

The parameters used in the Intermolecular Lennard-Jones potentials are:

.

| **Lennard−Jones** | **ε (eV)** | **σ (Å)** |
| --- | --- | --- |
| Ca−O_w_ | 0.00095 | 3.35000 |
| O_w_−O_w_ | 0.00674 | 3.16549 |

The parameters used in the Intermolecular Harmonic bond potentials are:

| **Bond** | ***k* (eV Å^−2^)** | ***r*_0_ (Å)** |
| --- | --- | --- |
| C−O | 35.90 | 1.313 |
| O_w_−H_w_ | 45.93 | 1.012 |

The parameters used in the Intermolecular harmonic angle potentials are:

| **Angle** | ***k* (eV rad^−2^)** | **θ_0_ (deg)** |
| --- | --- | --- |
| O−C−O | 12.000 | 120.00 |
| H_w_−O_w_−H_w_ | 3.291 | 113.24 |

The parameters used in the Intramolecular improper potentials are:

| **Improper** | ***k*_2_ (eV Å^−2^)** | ***k*_4_ (eV Å^−4^)** |
| --- | --- | --- |
| C−O/O/O | 20.796 | 360.0 |

**Supplementary References**

1. Goodwin, A. L. *et al.* Nanoporous Structure and Medium-Range Order in Synthetic Amorphous Calcium Carbonate. *Chem. Mater.* **22**, 3197–3205 (2010).

2. Cobourne, G. *et al.* Neutron and X-ray diffraction and empirical potential structure refinement modelling of magnesium stabilised amorphous calcium carbonate. *J. Non. Cryst. Solids* **401**, 154–158 (2014).

3. Jensen, A. C. S. *et al.* Hydrogen bonding in amorphous calcium carbonate and molecular reorientation induced by dehydration. *J. Phys. Chem. C* **122**, 3591–3598 (2018).

4. Fernandez-Martinez, A., Kalkan, B., Clark, S. M. & Waychunas, G. A. Pressure-induced polyamorphism and formation of ‘aragonitic’ amorphous calcium carbonate. *Angew. Chemie* **125**, 8512–8515 (2013).

5. Soper, A. K. Empirical potential Monte Carlo simulation of fluid structure. *Chem. Phys.* **202**, 295–306 (1996).

6. Raiteri, P. & Gale, J. D. Water Is the Key to Nonclassical Nucleation of Amorphous Calcium Carbonate. *J. Am. Chem. Soc.* **132**, 17623–17634 (2010).

7. Wu, Y., Tepper, H. & Voth, G. Flexible simple point-charge water model with improved liquid-state properties. *J. Chem. Phys.* **124**, 24503 (2006).
